# Maillard Proteomics: Opening New Pages

**DOI:** 10.3390/ijms18122677

**Published:** 2017-12-12

**Authors:** Alena Soboleva, Rico Schmidt, Maria Vikhnina, Tatiana Grishina, Andrej Frolov

**Affiliations:** 1 Department of Biochemistry, St. Petersburg State University, Saint Petersburg 199034, Russia; st021585@student.spbu.ru (A.S.); m.vikhnina@spbu.ru (M.V.); t.grishina@spbu.ru (T.G.); 2 Department of Bioorganic Chemistry, Leibniz Institute of Plant Biochemistry, 06120 Halle, Germany; 3 Department of Pharmaceutical Chemistry and Bioanalytics, Institute of Pharmacy, Martin-Luther Universität Halle-Wittenberg, 06108 Halle, Germany; rico.schmidt@pharmazie.uni-halle.de

**Keywords:** advanced glycation end products (AGEs), bottom-up proteomics, glycation, glyoxalase, model synthetic peptides, plant glycation, post-translational modifications, proteomics

## Abstract

Protein glycation is a ubiquitous non-enzymatic post-translational modification, formed by reaction of protein amino and guanidino groups with carbonyl compounds, presumably reducing sugars and α-dicarbonyls. Resulting advanced glycation end products (AGEs) represent a highly heterogeneous group of compounds, deleterious in mammals due to their pro-inflammatory effect, and impact in pathogenesis of diabetes mellitus, Alzheimer’s disease and ageing. The body of information on the mechanisms and pathways of AGE formation, acquired during the last decades, clearly indicates a certain site-specificity of glycation. It makes characterization of individual glycation sites a critical pre-requisite for understanding in vivo mechanisms of AGE formation and developing adequate nutritional and therapeutic approaches to reduce it in humans. In this context, proteomics is the methodology of choice to address site-specific molecular changes related to protein glycation. Therefore, here we summarize the methods of Maillard proteomics, specifically focusing on the techniques providing comprehensive structural and quantitative characterization of glycated proteome. Further, we address the novel break-through areas, recently established in the field of Maillard research, i.e., in vitro models based on synthetic peptides, site-based diagnostics of metabolism-related diseases (e.g., diabetes mellitus), proteomics of anti-glycative defense, and dynamics of plant glycated proteome during ageing and response to environmental stress.

## 1. Introduction

**Figure 1:**
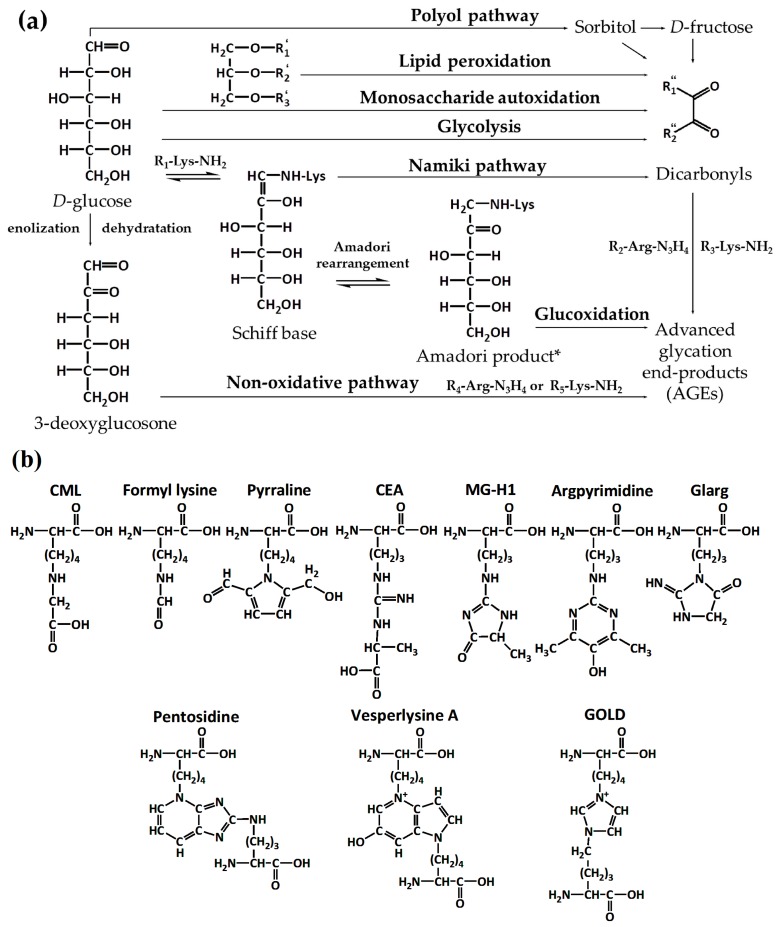
Pathways of early and advanced glycation (oxidative glycosylation [12], Namiki pathway [13], enolization [14], oxidative [15] and non-oxidative (enolization and dehydration stages are not mentioned) [16] degradation of early glycation products, polyol pathway [17] and lipid peroxidation [18] (**a**) and structures of major AGEs detected in vivo and in thermally processed foods (**b**)). R_1_, R_2_, R_3_, R_4_, R_5_, polypeptide chains; R_1_′, R_2_′, R_3_′, fatty acid residues; R_1_′′ = H, R_2_′′ = H for glyoxal; R_1_′′ = H, R_2_′′ = CH_3_ for methylglyoxal; R1′′ = H, R2′′ = C_4_H_9_O_3_ for 3-DG. * Ketoses form so-called Heyns products.

Glycation is referred to as the reaction of proteins with reducing sugars and dicarbonyl products of their degradation [1], and is often termed as Maillard reaction of proteins [2]. In its early steps, amino groups of polypeptides readily react with aldoses and ketoses yielding Amadori [3] and Heyns [4] compounds, respectively, via corresponding Schiff base intermediates (Figure 1a) [5]. The further degradation of these early glycation products yields a heterogeneous group of advanced glycation end-products (AGEs, Figure 1b), predominantly via glycoxidative pathway [6]. Alternatively, AGEs can be formed by reaction of α-dicarbonyls, such as glyoxal (GO), methylglyoxal (MGO) and 3-deoxyglucosone (3-DG), with lysyl and arginyl residues of proteins [7]. These highly-reactive intermediates are continuously generated in living organisms via oxidative degradation of sugars [7,8], lipid catabolism [9], polyol pathway [10] and non-enzymatic conversion of triosophosphate intermediates of glycolysis [11] (Figure 1a). Thus, the products of early and advanced glycation represent two derivative groups, differing in their structure, origin and physiological effects.

Indeed, during the last decades, deleterious effects of various AGEs (in contrast to early glycation products) in human organism were characterized in much detail [19,20,21,22], although protective and anti-oxidant activities were reported for some protein Maillard reaction products as well [23]. The most negative role of AGEs in human physiology is typically attributed to their pronounced pro-inflammatory effect, mediated by membrane or soluble receptors [24,25,26]. The most well-characterized representatives of this group are so-called receptors to advanced glycation end products (RAGEs), multiligand molecules, belonging to the immunoglobulin superfamily [27]. The surface ligation of RAGEs by AGEs results in activation of the transcription factor NF-κB, enhanced expression of adhesion molecules, and development of inflammation [27,28,29,30].

From the biochemical point of view, AGEs are the normal products of animal and plant metabolism, and their accumulation accompanies development and ageing, as well as multiple metabolic disorders [31,32]. However, in mammals, and, especially in humans, consumption with food is the principle root of AGE accumulation. Accordingly, adequate and reliable estimation of AGE contents is one of the most important problems to be solved by modern food chemistry, as it provides a direct access to risk assessment for possible inflammation-related diseases [33]. Although in earlier works, analyses of AGE contents were performed by enzyme-linked immunoassays (ELISA) [34,35], currently, chromatographic methods are recognized to be the most suitable for AGE analytics. Based on experimental data, snapshots of relative AGE contents in different foods can be obtained and organized in some systematic and well-accessible way (for example, see https://lemchem.file3.wcms.tu-dresden.de/).

In this context, due to outstanding robustness and reliability of this approach, exhaustive enzymatic hydrolysis of proteins and quantification of AGEs by liquid chromatography-tandem mass spectrometry (LC-MS/MS) became a gold standard in analysis of glycation products [36,37]. Enzymatic hydrolysis is usually performed as a multi-step procedure comprising a sequential treatment with several proteases (e.g., pronase E, leucine aminopeptidase, and carboxypeptidase Y, as proposed by Glomb and co-workers [36]). This procedure provides sufficient stability of imidazolone AGEs, as well as carboxymethylated and carboxyethylated derivatives [36]. However, when only acid- and temperature-stable derivatives are to be analyzed, conventional acid hydrolysis is sufficient to obtain reliable results [38]. The analysis of hydrolyzates typically relies on reversed phase chromatography (RPC) with or without application of ion pair (IP) reagents, and on-line mass spectrometric [37] or fluorescence detection [36]. To increase sensitivity of analysis, and to improve chromatographic behavior of analytes, different derivatization strategies can be applied [39,40,41,42]. Analysis of protein hydrolyzates can be complemented by profiling of free glycation adducts [31], that might provide a deeper insight in catabolism of glycated proteins. As was mentioned above, triple quadrupole (QqQ) instrumentation and tandem mass spectrometry (MS/MS) in a multiple reaction monitoring (MRM) mode is the most widely spread technique for analysis of glycation adducts [43]. However, ion trap (IT) [44] and quadrupole-time of flight (QqTOF) [45] mass analyzers can be employed as well. Absolute quantification of individual glycation adducts typically relies on standard isotope dilution [46] or standard addition techniques [36]. Although the latter approach requires higher sample amounts and longer analysis times, it delivers precise and reproducible results with fewer costs [36]. To get a deeper insight in mechanistic aspects of the Maillard reaction, analysis of glycation adducts can be complemented by analysis of their precursors, i.e., carbonyl compounds—carbohydrates [47] and α-dicarbonyls [48,49,50].

Although LC-MS/MS of glycation adducts is an excellent tool for comparison of different AGE sources or physiological states, this approach does not deliver any information about modified proteins and exact affected sites therein. However, as both formation and degradation of glycation products, at least to some extent, is dependent from protein sequence and structure [51,52,53], this information is strongly mandatory for understanding of the changes in protein functionality, related to glycation. In this context, proteomics is a powerful analytical tool, giving a direct access to identification and quantification of individual glycation sites.

Therefore, in the first part of this review we comprehensively discuss existing mass spectrometric techniques used for characterization of glycated proteins and promising in food safety applications and clinical diagnostics. Further, in the second part of this work, we address the novel trends in the study of glycated proteome: (i) model synthetic peptides as the tools of proteomic research; (ii) individual glycation sites as prospective clinical biomarkers; (iii) proteomics of anti-glycative defense; and (iv) glycation of plant proteins during ageing and under environmental stress conditions.

## 2. Part 1. Probing the Structure of Glycated Proteins by Mass Spectrometry

Analysis of post-translational modifications (PTMs) in proteins is a challenging task. Indeed, due to a high variability in modification levels at individual amino acid residues, it requires high dynamic range and sensitivity of MS instrumentation [54]. Thereby, due to a higher numbers of potential reactive sites, the patterns of non-enzymatic modifications are typically more complex, in comparison to those formed by enzyme-dependent mechanisms [55]. Finally, in comparison to other non-enzymatic modifications, glycation brings further challenges: (i) formation of isomeric products (e.g., glucose-derived Amadori and fructose-derived Heyns compounds) [51,56,57,58]; (ii) extremely high heterogeneity of appearing AGE structures [32]; (iii) occurrence of multiple diverse modifications in one protein molecule [59,60]; and (iv) even alternative modification of the same amino acid residue [32]. Hence, only limited characterization of protein glycation is possible on the level of intact protein, whereas comprehensive evaluation of modification patterns typically requires enzymatic digestion and tandem mass spectrometric analysis of resulted mixtures of proteolytic peptides.

### 2.1. Analysis of Intact Proteins

Analysis of intact proteins might be useful in estimation of an overall glycation load within a certain population of molecules [61]. For such experiments, however, resolving power of a mass spectrometer plays a crucial role [62]. Therefore, time of flight (TOF) mass analyzers in combination with matrix assisted laser desorption/ionization (MALDI) and electrospray ionization (ESI) are successfully applied to analysis of biomolecules since late 1980s [63].

#### 2.1.1. MALDI-TOF-MS of Intact Glycated Proteins

As was shown in the early works of Lapolla’s and Boratynski’s groups [64,65], formation of Amadori compounds can be clearly seen in MALDI-TOF spectra by characteristic shifts in molecular weights of target proteins and, hence, *m*/*z* of corresponding MS signals (e.g., +162 *m*/*z* for *N^ε^*-(fructosyl)lysine moiety). Indeed, even a relatively low resolution, achieved in the linear mode, allows determination the number of attached sugar moieties [66,67]. In the first line, it could be observed with in vitro glycated bovine serum albumin (BSA) [65,68,69,70,71], bovine pancreatic ribonuclease [68], and lysozyme [72]. Later, this concept was extended to hemoglobin (HbA) [73], α-lactalbumin (α-La) and β-lactoglobulin (β-Lg) [74], human serum albumin (HAS) [75], γB-crystallin [76], ribonuclease A (RNase A) [77], and horse myoglobin [78]. Recently, using MALDI-TOF-MS, Chaudhury et al. described in vitro modification of γB-crystallin with one hexose moiety, and reported formation of dimeric cross-links (as confirmed by polyacrylamide gel electrophoresis is sodium dodecyl sulfate (SDS-PAGE) and size-exclusion chromatography) upon the incubation for 60 days at 37 °C. According to the authors, it could impact in development of diabetic cataract in mammals [76]. In contrast, depending from glycation agent used, one RNase A molecule could react with 5–15 sugars equivalents, that resulted in complete inactivation of the enzyme and disruption of its interaction with ribonuclease inhibitor (RI) and DNA [77]. In vitro glycation of a total hemoglobin preparation revealed different glycation levels for individual variants, that resulted in a complex glycation profile [73]. Similarly, Pischetsrieder and co-workers demonstrated formation of hexose and lactose adducts of whey proteins during their heating (60 °C) in the model system mimicking lactose-free milk (α-La 1.3 g/L, β-Lg 3.2 g/L) in presence of glucose, galactose (both 22.0 g/L), and lactose (5.1 g/L) in phosphate-buffered saline [74].

In the next logical step, the described analytical strategy was successfully transferred to in vivo glycation systems [72,79,80,81,82,83]. Apparently, the changes in overall protein glycation status can be potentially used as diagnostically valuable markers. Thus, Lapolla and co-workers compared well- and bad-controlled type 2 diabetes mellitus (T2DM) patients with normoglycemic controls in terms of the number of fructosamine residues attached to HSA [66] and hemoglobin [84] in blood samples. Remarkably, for analysis of glycated hemoglobin, MALDI-TOF-MS provided a much higher sample throughput in comparison to conventional high-performance liquid chromatography (HPLC)-based protocols [79,85]. Recently, this diagnostic approach was extended to mitochondrial proteins from peripheral blood mononuclear cells [86]. Analogously, early glycation was characterized in placenta protein of T2DM patients [87], whereas formation of albumin-bound AGEs was addressed in uremia [88]. In all cases, T2DM was associated with a higher numbers of glycated adducts and a higher abundance of accordingly modified proteins.

A surface-enhanced laser desorption/ionization mass spectrometry (SELDI-MS), relying on selective affinity-based retention of specific molecules on an affinity chip, integrated with MALDI target, represents a promising alternative to gel-based techniques [89], and is mostly used for analysis of low molecular weight proteins [90]. Recently, Nedic et al. reported an application of SELDI to the analysis of serum fractions obtained by boronic acid- and lectin-affinity chromatography [91]. SELDI was also successfully used for characterization of glyoxal-derived modifications of bovine erythrocyte superoxide dismutase [92], for identification of inflammatory biomarkers, and for characterization of innate immunity in atrophic nonunion fracture [90]. The main advantage of this technique is high sensitivity, i.e., the ability to detect analytes, present in rather low concentrations [93]. It can be achieved by decreasing noise intensity due to additional purification of immobilized proteins prior to MS analysis [91].

#### 2.1.2. ESI-MS of Intact Glycated Proteins

Electrospray ionization (ESI) is another soft ionization technique, routinely applied to determination of protein molecular weights [94]. Under acidic conditions, used for ESI-MS in positive ion mode, proteins are polycations, and their ESI mass spectra are characterized with abundant charge series [95]. Deconvolution of charge series (i.e., complete resolving of individual adducts with different charge) is the prerequisite for correct determination of molecular weight [94]. Therefore, ESI-MS experiments with intact proteins rely on high resolution mass spectrometry (HR-MS) and instruments with a high resolving power, i.e., quadrupole-time of flight (QqTOF) [61,96], furrier transform-ion cyclotron resonance (FT-ICR) [97], and Orbitrap-based [98] mass analyzers. Thereby, samples can be injected in the mass spectrometer without separation (so-called flow injection analysis, FIA) [61,97,99,100,101], or after separation, which can be performed off-line or on-line [102,103,104,105].

Generally, FIA is applicable to simple mixtures or in vitro incubations with individual proteins [61]. For example, Stefanowicz and co-workers used this approach for monitoring of lysozyme glycation at 50 °C [100,106]. Further, the combination of deuterium–hydrogen exchange (DHX) and mass spectrometry (MS) was successfully applied to address the influence of glycation on high pressure denaturation (HPD) of proteins [97]. However, complex samples require additional affinity enrichment [102] or off-line pre-fractionation, which can rely, for example, on cation exchange chromatography (EXC) [102,105,107]. Individual fractions can be concentrated under reduced pressure and analyzed by ESI-MS operated in positive ion mode [102,104,105]. Remarkably, for highly abundant proteins, such as hemoglobin and albumin, ESI-MS analysis of biological samples can be performed without separation. Indeed, although ESI-MS is highly prone to matrix effects [108], reliable quantification of such proteins can be achieved. For example, according to Roberts et al., matrix effects can be effectively minimized (up to the degree compatible with acquisition of MS data) by a 500-fold dilution of sample in an acidic denaturing solvent [99]. Alternatively, protein mixtures can be separated by reversed-phase high performance liquid chromatography (RP-HPLC) on C4 columns [109] coupled to mass spectrometer on-line. Analogously, capillary zone electrophoresis (CZE) was applied to separation of individual HSA forms including native, oxidized, and glycated species [101], whereas microfluidic capillary electrophoresis coupled on-line to ESI-MS is a promising tool for determination of glycated hemoglobin and albumin in human blood [96].

Summarizing, although analysis of glycation on the level of intact protein represents a promising tool in medical diagnostics, this approach delivers only general information about the overall glycation levels of major proteins. Thus, it does not provide any insight in specific affected amino acid residues in individual polypeptides. This information, however, is required for understanding the mechanisms behind disease pathogenesis [96,99,110] and drug activity [109]. Therefore, analysis of specific glycation sites seems to be a promising strategy to understand the mechanisms of protein glycation in vivo and its biological role. In this context, proteomics is the methodology of choice to achieve these aims.

### 2.2. Proteomics Approach in Glycation Research

Proteomics aims qualitative and quantitative characterization of all proteins represented in certain cell, tissue, or organism [111,112,113]. Thus, it provides annotation of generated MS signals to individual proteins, and, if necessary, quantitative assessment of signal intensities and abundances of related analytes either on the absolute or relative basis [112,114,115,116]. Sequence annotation of individual proteins can rely on: (i) tandem mass spectrometric (MS/MS) analysis of protein quasi-molecular ions (top-down approach) [117,118,119]; or (ii) limited hydrolysis of proteins or their complex mixtures with subsequent sequence assignment of resulted cleavage peptides by tandem mass spectrometry (bottom-up approach) [83,120,121].

#### 2.2.1. Top-Down Proteomic Strategy

Top-down proteomics (TDP) typically relies on isolation of full protein quasi-molecular ions by trapping techniques on the level of mass analyzer, with their subsequent MS/MS analysis. Thus, the whole procedure does not include an enzymatic digestion step [122,123,124]. This workflow allows identification and quantification of individual post-translational modifications (PTMs), unique proteoforms (e.g., proteins with post-translational modifications or having slightly different sequence), sequence variations, positional isomers, and specific products derived by alternative splicing [111,118,125,126,127]. Most often, proteins are ionized by ESI and trapped in a Fourier transform-ion cyclotron resonance (FT-ICR) or quadrupole ion trap (QIT) mass analyzers [117,128] with fragmentation, based on electron capture dissociation (ECD) or electron transfer dissociation (ETD) [119,126,129]. Less commonly, top-down experiments are based on MALDI-TOF/TOF analyzers and post-source decay fragmentation [127]. Generally, ESI is a preferred technique, since it produces multiply-charged precursor ions for more efficient dissociation of large protein ions and provides more MS/MS options than MALDI which mainly produces singly-charged species.

To a large extent, top-down proteomics can be considered as a strategy, complementary to the conventional bottom-up approach, especially useful in analysis of small proteins with a limited numbers of specific sites of protease cleavage and present in high concentrations [116,119]. Therefore, pre-separation by RP-HPLC or gel electrophoresis is conventionally applied [118,119]. Moreover, selection of protein solubilization conditions, compatible with purification, separation and ionization in MS source, is rather challenging [127], and might require application of detergents [127,130], concentrated acids [128], or use of sophisticated multi-dimensional separation techniques such as gel-eluted liquid fraction entrapment electrophoresis (GELFrEE) coupled to LC-MS/MS [124,126,130].

Generally, TDP is the method of choice for identification, characterization and quantification of individual proteoforms as potential clinical biomarkers [118,126]. For example, TDP is able to distinguish glycated isoforms of HSA, HbA and apolipoprotein I (Apo-I) from corresponding unmodified analogs [118], that is critically important for a reliable distinguishing of T2DM patients from normoglycemic individuals. It can be efficiently applied in a “single drop” LC-MS/MS analysis of multiple biomarkers of hyperglycemia, oxidative stress, and cardiovascular risks [118].

#### 2.2.2. Bottom-Up Proteomic Strategy

In contrast to TDP approach, the bottom-up proteomic (BUP) strategy can be applied to protein mixtures of any composition and complexity [114]. It relies on: (i) separation of proteins; (ii) limited proteolysis; (iii) separation of resulted cleavage peptides; (iv) their identification by tandem mass spectrometry (MS/MS); and (v) annotation of individual protein sequence tags [123,131,132,133]. In application to sugar-modified proteins, BUP provides detailed information about glycoprotein profile and gives an access to specific mapping of glycosylation sites [134]. As this methodology typically relies at least on two separation steps (on a protein and/or peptide level prior to separation by mass-to-charge ratio), it allows higher proteome discovery rates and sensitivities in comparison to TDP [114,123,133,135,136]. However, due to a high specificity of endoproteases used for digestion, sequence coverage of individual proteins might strongly depend from their sequences [137,138,139]. Moreover, only relatively small portion of identified proteolytic peptides is represented by unique sequence tags, whereas the most of them can be annotated to several proteins [137,138,140,141]. Besides, degradation of labile PTMs during proteolysis (personal observation of the authors) and strong matrix effects represent serious challenges in BUP [142,143]. To some extent, these complications can be overcome by so-called middle-down approach, i.e., MS/MS analysis of proteolytic fragments with the molecular weights of 3–20 kDa [136], obtained by protein hydrolysis with highly-specific endoproteases (characterized with a low number of unique cleavage sites, recognized in substrate polypeptides, e.g., GluC or AspN) [67,123,136].

##### Limited Enzymatic Proteolysis

In this context, correct selection of proteolytic enzymes is critical for success of the whole BUP experiment. Accordingly, a wide range of proteases of various specificity were proposed during the last decade (Table 1): trypsin [144,145], chymotrypsin [146,147], LysC [137,148], AspN [67,74], GluC [149,150], endopeptidase Arg C [137], pepsin [151], proteinase K [152], and papain [153]. Due to its moderately high specificity (C-terminally from K and R residues) and convenient size of resulting hydrolytic peptides (0.5–3.0 kDa) [154], trypsin remains the most widely used protease [155]. Commercially produced proteomics grade trypsin is chemically modified, to inhibit its autocatalytic activity and to ensure high specificity of cleavage [155]. Therefore, tryptic digestion typically results in relatively high sequence coverage rates (up to about 90% for HSA) [148], which can be further increased when trypsin is combined with other proteases [83,114].

It is important to mention that PTMs in general and glycation in particular are known to reduce efficiency of enzymatic proteolysis [139,161]. Thus, tryptic digestion of glycated HSA yielded only one third of the total number of tryptic peptides, obtained with unglycated protein under the same conditions [139]. Thereby, the percentage of peptides containing missed cleavage trypsin sites increased [139]. Interestingly, according to our experience, proteomic grade trypsin of different producers has different reactivity towards modified lysyl and arginyl residues in proteins. Thus, Promega trypsin, often used in quantitative proteomic applications [162,163], is much more reactive than the product of Serva, typically applied in discovery proteomics [164,165], and yields a less number of missed cleavage peptides. Moreover, according to our observations, Promega trypsin results in cleavage of Amadori moieties and, therefore, reduced recoveries of glycated peptides.

**Figure 2:**
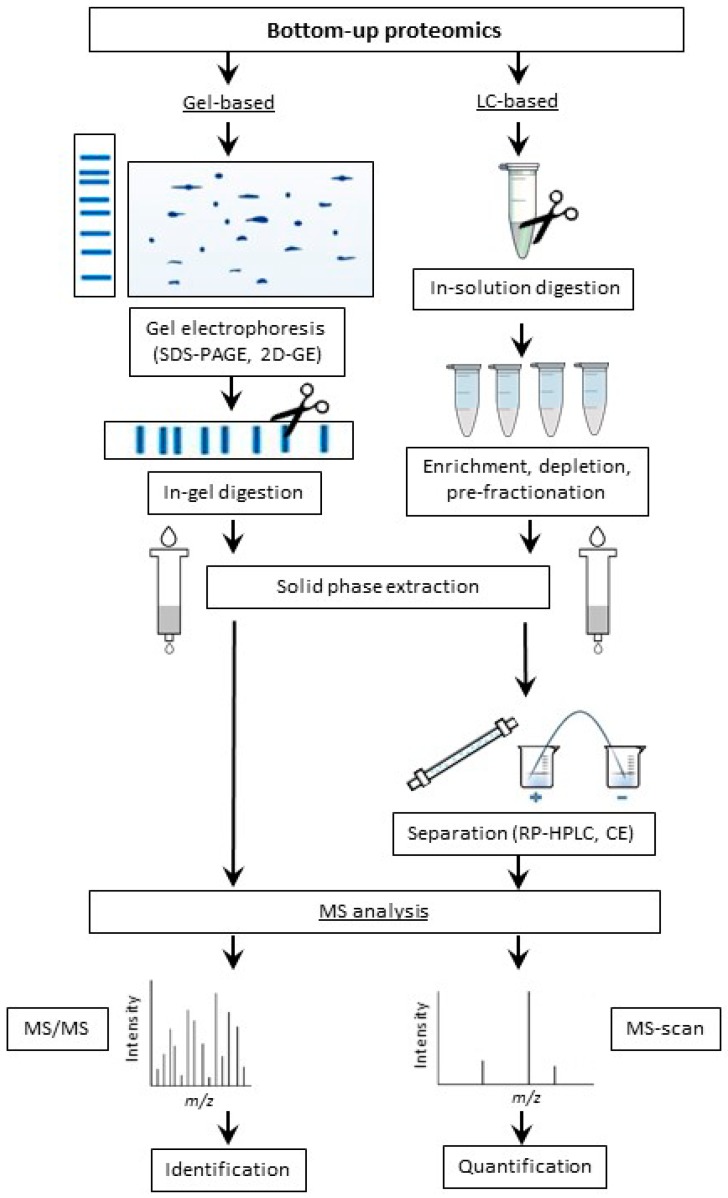
The overview of the gel- and LC-based workflows in bottom-up proteomics. The scissors denote enzymatic proteolysis.

Most often, BUP relies on two main workflows, i.e., gel- and liquid chromatography (LC)-based strategies (Figure 2) [2,166]. In terms of the gel-based approach, protein mixtures can be separated by polyacrylamide gel electrophoresis in sodium dodecyl sulfate (SDS-PAGE) [87,167,168] or by two-dimensional gel electrophoresis (2D-GE) [169,170,171,172,173], with subsequent digestion of the proteins representing individual electrophoretic zones followed by MS or/and MS/MS analysis of resulted hydrolysates. In contrast, LC-based approach relies on limited enzymatic proteolysis of complex protein mixtures (such as cell lysates or tissue extracts) with subsequent separation of resulted hydrolytic peptides by RP-HPLC [52,174,175] or capillary electrophoresis (CE) [176,177] with on-line mass spectrometric detection [114,177].

The gel-based strategy typically employs MS-compatible visualization techniques. Thus, most often, it relies on Coomassie brilliant blue dye [171,172,173], whereas improved sensitivities and specificities of analysis can be achieved by silver staining (without addition of glutaric aldehyde) [178,179] and application of sample-specific fluorescent dyes, e.g., cyanines 2, 3 and 5 (Cy2, Cy3, and Cy5, respectively) as a part of the difference gel electrophoresis (DIGE) workflow [180]. For identification, the visualized electrophoretic zones (representing individual proteins or relatively simple protein mixtures) are excised, destained, and immobilized proteins are dehydrated by acetonitrile [171,181] prior to in-gel reduction of disulfides, alkylation of resulted sulfhydryls [87], and enzymatic in-gel digestion with subsequent identification of proteolytic peptides by MALDI- [167] or LC-ESI-MS [182].

Although in-solution digestion seems to be an easy procedure, finding a compromise between the completeness of hydrolysis and compatibility of the procedure with mass spectrometric analysis, might be a challenging task [114]. Indeed, to achieve a quantitative cleavage of all possible endoprotease sites (also located in hydrophobic parts of polypeptide chain), the protein sample needs to be completely solubilized and unfolded. For this, addition of detergents and chaotropic agents (e.g., urea and thiourea) is strongly mandatory [114,175]. After the completion of hydrolysis, urea and thiourea can be easily removed from the digestion mixture by reversed phase solid phase extraction (RP-SPE) [52]. However, conventional detergents, such as SDS and Triton X100, are efficiently retained on reversed phase, co-elute with proteolytic peptides, and interfere with their detection, representing the most serious challenge in LC-based proteomics [183].

Fortunately, this limitation can be overcome by application of degradable detergents, recently introduced in proteomics laboratory praxis [114]. Such compounds can be destroyed after enzymatic hydrolysis and removed by SPE afterwards. Currently, several commercial products, delivering reliable and reproducible results are available: anionic acid labile surfactant (AALS) from Progenta [53,175], acid labile RapiGest SF Surfactant from Waters Corporation [184,185]. Additionally, acid cleavable detergents ProteaseMax (Promega) [186], PPS Silent Surfactant (Expedeon) [187], and Invitrosol (Invitrogen) [188] might be used in analysis of post-translationally modified proteins. Interestingly, application of boronic acid affinity chromatography (BAC) as an orthogonal separation technique prior to nanoRP-HPLC allows application of non-degradable detergents, such as SDS [115,174,189]. Indeed, SDS is not retained on the affinity column and is quantitatively washed out during the BAC procedure, not interfering with subsequent MS analysis [189].

After addition of a buffer containing chaotropic agent and detergents (often referred to as shotgun or lysis buffer) and complete solubilization of the sample, reduction of disulfides and alkylation of sulfhydryls is performed as described above [114]. Thereby, *tris*-(2-carboxyethyl)-phosphine hydrochloride (TCEP), β-mercaptoethanol, dithiothreitol (DTT) or dithioerithritol (DTE) serve as reducing agents, whereas alkylation usually relies on iodoacetamide [172,174]. Alternatively, maleimide derivatives [190,191], acrylamide [192], 4-vinylpyridine [193,194], iodoacetic acid [195,196], and chloroacetamide [197,198] can be used as alkylation agents. It is important to keep in mind that the pH of the digestion buffer needs to be adjusted to the optima of corresponding enzymes (Table 1), and its molarity needs to ensure a complete buffering of the sample. For example, 50–100 mmol/L ammonium bicarbonate buffers with pH 8.0 are ideal for in-solution tryptic hydrolysis [155,167,174].

During the last decade, multiple approaches to increase digest completeness in parallel to minimization of proteolysis times were introduced. Thus, an ultrafast tryptic digestion procedure, requiring only five minutes directly prior to LC-MS/MS experiments, was proposed by Wang et al. [121]. An alternative approach relies on a hydrolysis reactor containing immobilized trypsin. In this design, the enzyme can be conjugated to magnetic nanoparticles, modified with a polyamidoamine dendrimer, via a DNA linker [199]. This setup allowed digestion of glycated hemoglobin with sequence coverage of up to 88%, with just a negligible loss of enzymatic activity over a period of two weeks.

##### Application of Gel-Based Proteomics in Maillard Research

Currently, two-dimensional gel electrophoresis (2D-GE) is the “working horse” of the gel-based proteomics [114]. Thereby, individual electrophoretic zones typically contain only few proteins. Therefore, corresponding enzymatic digests demonstrate relatively low complexity [2,167,170], and can be analyzed by mass fingerprint analysis (sometimes also referred to as peptide mapping) using MALDI-TOF-MS in a reflectron mode [87]. This MS technique is traditionally used for sequence confirmation of individual proteins and verification of predicted PTM patterns [114]. In particular, glycation sites can be assigned by a comparison with databases containing specific mass increments, characteristic for individual products, as was exemplified in experiments with placenta homogenates from pregnant women with gestational diabetes mellitus (DM) [87]. The same strategy can be applied to in vitro glycation systems. Thus, Pischetsrieder and co-workers described an integrated SDS-PAGE/MALDI-TOF-MS approach for milk samples heated during different times [167]. It allowed assignment and relative quantification of oxidation and glycation sites in β-lactoglobulin. The same approach could provide insight in the protein damage (e.g., deamidation and lactosylation) occurring in ultra-heated milk during storage [169] and identification of lactose-derived modification sites in α-lactalbumin [170]. Further, by this approach, Calvano et al. confirmed modified milk proteins and peptides as the indicators of powdered milk in food [171].

However, despite of ease in handling and a high-throughput of MALDI-TOF-MS, it might be insufficient for comprehensive and reliable identification of all proteins co-migrating in SDS-PAGE, especially, when it is not pre-faced by isoelectrofocusing [87]. Therefore, MALDI-TOF/TOF instrumentation, relying on post-source decay and collision-activated dissociation (CAD) fragmentation capabilities, provides much more reliable tandem mass spectrometric data [200].

##### Application of LC-Based Proteomics in Maillard Research

The LC-MS-based bottom-up techniques for analysis of protein mixtures are usually referred to as “shotgun proteomics” [114]. Thereby, the proteins are digested without any separation, which is applied only at the step of proteolytic peptides. Identification typically relies on comparison of all acquired MS/MS spectra with in silico calculated proteome databases, with consideration of enzyme specificity [114,201,202,203]. Typically, shotgun techniques have higher analytical resolution in comparison to those based exclusively on protein separation. Indeed, even such a powerful method as 2D-GE in combination with highly-sensitive visualization techniques yields maximally about 1500 individual signals (so-called “spots”, each of which can represent, however, several non-separated proteins) [204]. In contrast, RP-HPLC and ultra-high performance liquid chromatography (UHPLC) techniques provide much better resolution and higher identification rates. Thus, recently, Köcher and co-workers reported 2761 proteins identified in one shotgun experiment [205].

To achieve the highest possible sensitivity of analysis, LC separation typically relies on nano-flow RP-HPLC/UHPLC on C18 silica-based or polymeric columns with the internal diameters (i.d.) of 75–100 μm [137,146]. In classical shotgun experiments, column effluents are directly transferred in an ESI source of a highly sensitive hybrid QqTOF [206], QqQ [207,208], QqQ-LIT [209], IT-TOF [210], LIT [211], FT-ICR [212], LIT-Orbitrap [213], Q-Orbitrap [214] and Q-Orbitrap-qIT [215] mass spectrometers. Off-line LC-MS coupling, e.g., by transfer of effluent fractions to a MALDI target with a subsequent MALDI-TOF/TOF-MS analysis, can be considered as an alternative to conventional shotgun experiments [216].

Hyphenated LC-based techniques relying on data-dependent [32] and data-independent [217] acquisition algorithms (DDA and DIA, respectively) were successfully employed in glycation research, and proved to be much more powerful tools in comparison to gel-based approaches [156,218,219]. For example, glycation sites in HSA of DM patients were successfully identified by LC-IT-TOF-MS [83]. Analogously, glycation patterns of heterologically expressed recombinant HSA were addressed by nano-scaled liquid chromatography (nanoLC)-QqTOF-MS [146]. Remarkably, the shotgun approach can be applied to analysis of peptide mixtures, obtained by in-gel digestion of individual electrophoretic zones. For example, using nanoRP-HPLC-ESI-QqTOF- and ESI-QqQ-MS, Marvin et al. identified about 40 sites in six milk proteins and proposed α-lactoalbumin as a marker of lactosylation in milk [172].

Similar to gel-based proteomics, LC-based approach can allow both identification and quantification, or can be focused only on one of these aspects. When identification of protein glycation sites is the main scope of research (so-called “discovery proteomics”), the overall analytical resolution, i.e., the total number of identified features, is of the major importance. In the most easy and straightforward way it can be increased by application of longer columns and gradients times, providing high peak capacity [220]. However, the coverage of modified proteome can be further increased by implementation of additional separation procedures, introduced in the general proteomics workflow as enrichment, depletion, or pre-fractionation steps [137,221,222,223]. Such multi-dimensional workflows allow reduction of matrix effects and, hence, provide better sensitivity [108]. Moreover, when the DDA approach is used, decrease of sample complexity allows overcoming of so-called “undersampling effect”, i.e., limitation of the identification event number by the duration of instrument duty cycle [224,225].

**Figure 3:**
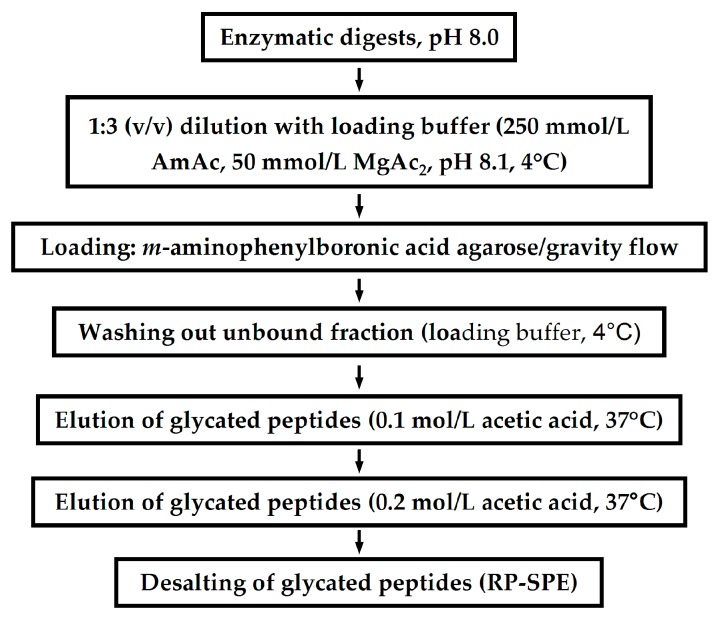
Enrichment of glycated (Amadori) peptides by BAC [127]. The experimental workflow relies on *m*-aminophenylboronic acid-agarose, filled in polypropylene columns, and gravity flow design.

For early glycated species, enrichment by BAC is the method of choice [226,227]. Although this procedure can be applied both to protein mixtures and products of their enzymatic hydrolysis, enrichment on the level of digests was shown to be advantageous [52,219]. During the recent decade, the power of this method was confirmed with in vitro glycated proteins [219,222,228], human plasma [115,137,174,189] and even plant tissues [32]. To achieve higher recoveries of glycated peptides, two-step elution with warm (37 °C) aq. acetic acid (0.1 and 0.2 mol/L) proved to be most suitable (Figure 3) [219]. After a solid phase extraction (SPE)-based pre-cleaning step, samples are typically freeze-dried, reconstituted in 3% acetonitrile in 0.1% (*v*/*v*) aq. formic acid, and analyzed with nanoU(H)PLC-ESI-MS [115]. Interestingly, to address a low-abundant part of the glycated proteome, BAC-based enrichment can be efficiently combined with immunoaffinity depletion technique [114,137] and selective precipitation of target proteins [83,114,227]. As the most generalized and characteristic example, can serve the work of Zhang et al. (2011), who depleted human plasma for the twelve most highly abundant proteins prior to the enzymatic digestion, multi-dimensional LC separation and MS analysis, which resulted in identification of thousands glycation sites [52].

Due to a high structural heterogeneity, enrichment of AGEs is a challenging task. In the most easy and straightforward way, it can be accomplished for individual AGE classes by affinity chromatography on immobilized specific antibodies (immunoaffinity approach) or immobilized RAGEs [2,229]. As proteins, modified with *N^ε^*-(carboxymethyl)lysine (CML) and pentosidine, bind to sepharose 4B-linked lysozyme [229], appropriate methods can be established as well. Alternatively, AGEs and advanced lipoxidation end products (ALEs) can be enriched by magnetic beads functionalized with the RAGE VC1 domain [230]. Recently Prassana et al. reported enrichment of AGE-modified peptides containing CML, *N^ε^*-(carboxyethyl)lysine (CEL), 1-(4-amino-4-carboxybutyl)-2-imino-5-oxo-imidazolidine, glyoxal-derived hydroimidazolone (Glarg), *N^δ^*-(5-methyl-4-oxo-5-hydroimidazo-linone-2-yl)ornithine, methylglyoxal-derived hydroimidazolone 1 (MG-H1), tetrahydropyrimidine (THP), 3-deoxyglucosone-derived hydroimidazolone (3-DG-H), and imidazolone B by immobilized metal affinity chromatography Cu(II)-IMAC [231].

Pre-fractionation is another strategy to increase analytical resolution in PTM-proteomics [232]. Typically it relies on separation techniques, orthogonal to RPC [233]. In the most common way, it can be accomplished by EXC, performed either off-line or on-line to the second dimension (RP-HPLC). Moreover, as was demonstrated by Metz and co-workers, this technique can be successfully combined with immunoaffinity chromatography, RPC and BAC in one analytical workflow. It provided excellent identification rates for glycation sites (3742 proteins represented by 7749 Amadori peptides) [52]. Alternatively, hydrophilic interaction liquid chromatography (HILIC) can be used as a pre-fractionation step [165], that was recently applied to identification of AGE-modified sites in human plasma [234] and *Arabidopsis thaliana* proteins [223].

It is important to note, that fractionation can be also performed on the level of mass analyzer. This approach, usually termed as gas phase fractionation (GPF), relies on multiple measurements of the same sample using different *m*/*z* ranges, defined by multipole devices [19,235]. During the last decade, this technique proved to be an efficient tool in discovery proteomics: it increases the numbers of identified peptides and, hence, sequence coverage of annotated proteins [19,115,223]. To increase the number of glycated peptides, identified in each experimental group, all positive hits can be cross-annotated between the samples. In the most easy way it can be done by exact *m*/*z*, charge and t_R_ [53]. A more sophisticated approach relies on time-based inclusion lists, based on targeted discovery experiments [165].

In discovery proteomics, efficiency of fragmentation in DDA or DIA experiments directly affects protein identification rates and coverage of glycated proteome [236]. Although most of the commercially produced mass spectrometers have only one fragmentation capability CAD, different combinations of CAD (performed either in trap or RF-only quadrupole collision cell) with ECD and ETD, respectively [114] are available in new state-of-the-art instruments. This also provides higher quantification accuracy, access to complementary ion information and improved proteome coverage [237].

For early glycated proteins, CAD provides important structural information, which can be obtained in neutral loss, product and precursor ion experiments. Thus, Gadgil et al. identified 31 glycated lysyl residues in HSA by characteristic hexose-related neutral losses of 162 Da in MS/MS spectra of tryptic Amadori peptides [238]. Further exploration of such MS/MS spectra revealed intense signals, corresponding to losses of water and formaldehyde [239,240], which are not only diagnostic for Amadori or Heyns moieties, but can be also used for sequencing of proteolytic peptides [241]. Remarkably, corresponding immonium-related ions can serve as diagnostic fragments in specific precursor ion scanning experiments performed with characteristic signals of early and advanced glycation products [239,242,243]. Alternatively, other MS/MS techniques, such as neutral loss triggered MS^3^ (NLMS^3^) and multi-stage activation (MSA, i.e., sequentially applied CAD and ETD) were successfully employed for characterization of glycation in enzymatic digests [2,228]. Thereby, both NLMS3 and MSA experiments rely on characteristic water and formaldehyde neutral losses [244], or the loss of the whole glycation moiety [221]. Interestingly, comparison of NLMS3 and MSA revealed higher glycation discovery rates with the latter approach: for example, Pepaj et al. discovered 21 and 31 glycated peptides with these methods, respectively [228].

As ETD and ECD yield c and z ions exclusively by backbone fragmentation [114,240,245], these techniques are well-applicable to analysis of labile PTMs. Indeed, such modifications which remains unaffected under these conditions [114] and result in significantly higher identification rates in comparison to CAD [246]. Thereby, to suppress unmodified quasi-molecular ions and to change fragmentation patterns in favor of modified c and z-ions, double resonance (DR)-ECD was successfully applied. However, as the charge reduced species dominate during ionization, the fragmentation efficiency of ETD is lower in comparison to ECD. This can be, however, circumvented by the MSA approach [247].

Quantitative analysis of glycated peptides relies either on labeling or label-free techniques. Thus, ^18^O-labeling of HSA peptides was successfully applied for characterization of glycation dynamics [59,248] and early diagnostics of T2DM [249]. Another informative labeling technique relies on incubation with [^13^C_6_]glucose under the conditions mimicking in vivo glycation [131,151,221]. In terms of this approach, relative quantification relied on doublet signals representing in vivo glycation with [^12^C_6_]glucose and in vitro incorporation of [^13^C_6_]glucose [131,151,250]. Finally, standard isotope dilution techniques might rely on synthetic ^13^C,^15^N-labeled peptides, spiked to plasma samples for a high-throughput characterization of their glycated profiles by multiple reaction monitoring [189]. In contrast, label-free quantification approach is a fast and efficient technique to compare relative abundances of glycated proteins or individual glycation sites therein by intensities of corresponding peptide signals. This methodology is less cost intensive in comparison to labeling techniques, reliable and can be easily applied to analysis of low abundant peptides [114].

## 3. Part 2. New Prospectives in Maillard Proteomics

The late 1990s–early 2000s were the period of establishing principle glycation mechanisms [41,251,252,253,254] and glycation adduct patterns in foods [255] and clinical pathology [36,256]. Besides, the patterns of in vitro and in vivo glycation sites were characterized in multiple proteins, and even whole proteomes [52]. It was accompanied with establishing of highly-effective peptide synthesis workflows and development of new mass spectrometers (e.g., Orbitrap-based hybrids [215] and new generation of QqTOF instruments) and data acquisition algorithms (e.g., data-independent acquisition, known as MS^E^ [257] and SWATH [258]). Finally, high-throughput proteomic platforms, search engines and data-interpretation pipelines were introduces in this time [59,223]. Altogether, these factors played a crucial role in a rapid development of several new fields in protein glycation research, which are methodologically based on bottom-up proteomics techniques. In this section, we address some of them: (i) application of synthetic peptides as the models in glycation experiments; (ii) diagnostic approaches based on glycation at specific sites; (iii) proteomics of anti-glycative defense; and (iv) analysis of plant glycated proteome.

### 3.1. Synthetic Peptides as Model Systems in Maillard Proteomics

The knowledge about specific glycation sites in proteins is a pre-requisite for understanding their functional changes related to the Maillard reaction [259]. However, as individual proteins can contain dozens of potential glycation sites [60], and modification levels at each of them might be affected by protein sequence and structure [52,53], characterization of specifically modified amino acid residues in proteins is a challenging task. In contrast, although amino acid-based systems represent convenient and informative glycation models [260,261], they, however, do not consider inter-residue interactions. Obviously, for understanding of the mechanisms, underlying the product patterns, formed at individual sites, less complex models (considering, however, the neighboring residue effects) are required. In this context, synthetic peptides represent an ideal experimental approach to address the mechanisms of glycation, kinetics of AGE formation and degradation, as well as the influence of neighboring residues on these aspects. Obviously, by means of this tool, more reliable approximations on the protein level can be done. Therefore, the peptide-based approach is useful for establishing new analytical techniques, promising in medical diagnostics and food safety analysis [151,174,219,262].

The history of the peptide-based Maillard research comes back to the early 1990s, when Smith and Thornalley proposed the simplest dipeptide hippuryl-lysine model to monitor the degradation of Amadori products accompanied with formation of CML [263,264]. Later, longer peptides (representing artificial [265,266,267,268] and natural [266,269,270,271] protein sequences) were employed to address kinetic and mechanistic aspects of glycation adduct formation, structure of novel products [265,266,267,268,272], and influence of reaction conditions on AGE patterns [269,273,274,275]. To understand the mechanisms of AGE formation in such in vitro glycation model systems, and to dissect individual pathways of advanced glycation, analysis of peptide products can be complemented by quantification of carbohydrate [47] and α-dicarbonyl [276] intermediates. In such cases, analysis of carbohydrates relies on sequential derivatization with methoxyamine hydrochloride (MOA) and *N*-methyl-*N*-(trimethylsilyl) trifluoroacetamide (MSTFA), followed with GC-EI-MS relying on well-standardized methods [47], whereas the α-dicarbonyl patterns are typically addressed by liquid chromatography with ultraviolet detection (LC-UV), LS-MS or LC-MS/MS after derivatization of the α-dicarbonyl moieties with *o*-phenylenediamine (oPDA) [49,277].

**Figure 4:**
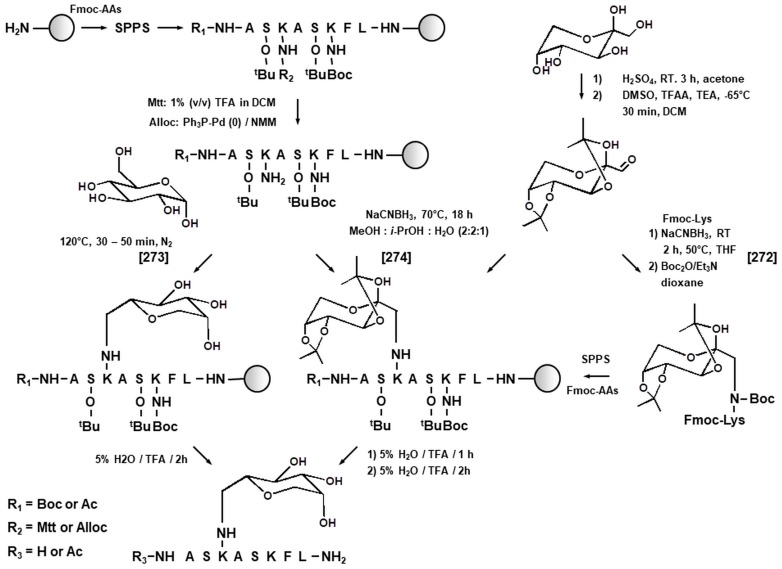
Synthesis of Amadori-modified peptides by global glycation approach and building block strategy. After selective deprotection of the site to be glycated, Amadori moiety can be introduced directly by incubation with reducing sugar [273] or via the Lobry de Bruyn reaction with acetonide-protected hexodiulose (2,3:4,5-di-*O*-isopropylidene-aldehydo-β-d-arabino-hexos-2-ulo-2,6-pyranose) in presence of cyanoborohydride in methanol-isopropanol-water mixture (2:2:1 by volume) [274]. Alternatively, glycated moiety can be introduced with an acetonide-protected *N^ε^*-Boc-*N^ε^*-fructosyl-*N^α^*-Fmoc-lysine building block [272].

A deeper insight in the pathways of the protein Maillard reaction can be obtained by means of model synthetic glycated peptides, used as the objects of kinetics studies and standards for structure elucidation [267]. Thus, Amadori peptides can be obtained by liquid phase [278,279] or solid phase [280] peptide synthesis. Due to its higher throughput and well-established robotized workflows, solid phase peptide synthesis (SPPS) seems to be advantageous. In the most direct way, glycation moiety can be introduced in a resin-bound peptide by global post-synthetic glycation after a specific cleavage of orthogonal protection group (typically allyloxycarbonyl or methyl trityl) at the ε-amino function of lysyl residue to be modified [281,282] (Figure 4). Thereby, derivatization might relay on direct glycation with reducing sugars, dissolved in DMF [281] or methanol [279]. Alternatively, glycation moiety can be introduced by the Lobry de Bruyn reaction with acetonide-protected hexodiulose (2,3:4,5-di-*O*-isopropylidene-aldehydo-β-d-arabino-hexos-2-ulo-2,6-pyranose) in presence of cyanoborohydride in methanol-isopropanol-water mixture (2:2:1 by volume) (Figure 4) [282]. Alternatively, protected hexodiulose can be reacted with a Fmoc-derivative of α-Boc-protected lysine [283], that gives an access to a building block strategy for the synthesis of glycated peptides [280]. Amadori- or Heyns-modified peptides can be easily purified by ion pair-reversed phase chromatography (IP-RP-HPLC) [284]. Analogously, AGE-modified peptides, containing the AGEs most abundant in human tissues and foods (CML, CEL, MG-H1, 2, and 3, Glarg, *N^δ^*-(carboxymethyl)arginine (CMA) and *N^δ^*-(carboxyethyl)arginine (CEA)) were synthesized by global post-synthetic derivatization [243] or building block strategy [23,285].

Synthesis of glycated peptides in high yields and purities gave an access to the mechanisms and kinetics of early glycation, Amadori degradation, and AGE formation [243,267]. Moreover, model reactions with defined sets of synthetic peptides provide a possibility to address the effects of individual neighboring residues on glycation rates and product patterns. Thus, histidyl imidazole groups and anionic residues, located in close proximity to the glycation site, catalyze Amadori rearrangement [51,286] and increase stability of resulting early glycation products [267]. Formation of arginine-derived hydroimidazolones and their hydrolysis products (CMA and CEA) also depends from the residues in the *i* + 4 position relative to the glycation site [268]. The effects of glycation on protein structure was addressed: the analysis of glycated α-helical peptides representing bovine serum albumin (BSA) sequence clearly indicated distortion of the helix, magnifying the impact of glycation on protein structure and, hence, potentially on their function [287]. On the other hand, a cross-linking AGE glyoxal-derived lysine dimer (GOLD) did not affect the structure of synthetic collagen fibrils [271]. It is important to mention, that synthetic peptide models represent ideal test-systems for probing potentially anti-glycative agents, which can be spiked to peptide-containing incubation mixtures with reducing sugars or α-dicarbonyls before assessment of Maillard reaction by electron spin resonance [288].

**Figure 5:**
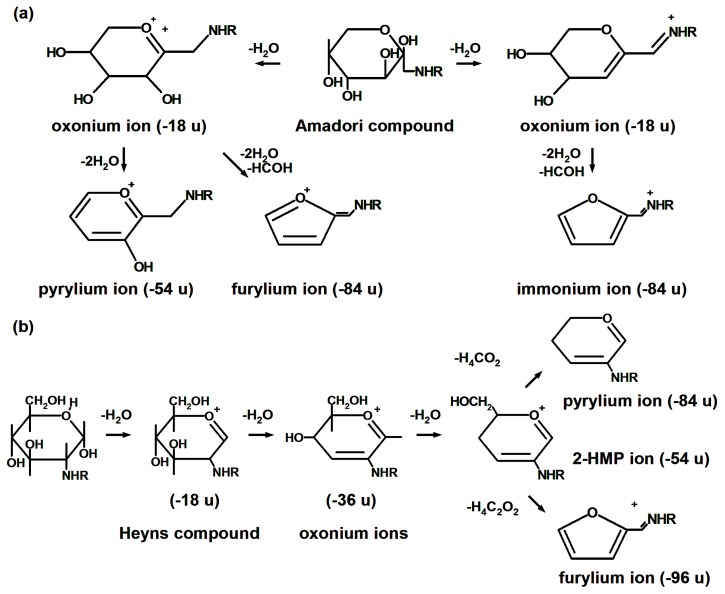
Tandem mass spectrometric (MS/MS) fragmentation patterns of: glucose-derived Amadori (**a**); and fructose-derived Heyns (**b**) peptides, represented by characteristic signals, corresponding to oxonium (-H_2_O and -2H_2_O), pyrylium (-3H_2_O) and furylium/immonium (-3H_2_O-HCHO) ions. The Heyns products can be distinguished by the presence of 2(hydroxymethyl)pyrylium (2-HNP) ion and specific mass increment of the Heyns-derived furylium signal (96 u).

Besides kinetic studies and pathway characterization, synthetic glycated peptides can be employed for development of new analytical techniques. In the first line, such peptides might be useful in interpretation of MS/MS fragmentation patterns, validation of quantitative methods, and establishing of new diagnostic approaches [219,239,289]. In this context, simple models with defined amino acid composition allow disclosing the pathways of their fragmentation under CAD conditions, which are rather complex due to simultaneous cleavage of the peptide backbone and sugar moieties [278]. Indeed, due to a relatively low energy of the bonds within carbohydrate moiety, the CAD-MS/MS spectra of glucose-derived Amadori peptides are strongly dominated by the sugar-related neutral losses of water and formaldehyde, represented in the spectra by oxonium, pyrylium and furylium ions (loss of 18/36, 54 and 84 u, respectively, Figure 5) [239]. It corresponds well to the fragmentation patterns observed at the amino acid level in experiments with free fructosamine-modified lysine [290]. Interestingly, glycation products, derived from isomeric aldoses and ketoses, can be distinguished by these signals: for example, fructose-derived Heyns products can be unambiguously identified by characteristic 2-hydroxymethylpyrylium, pyrylium, and furylium ions (−54, −84, and −96 u, respectively, Figure 5) [239]. Importantly, the fragment ion series are dominated by corresponding neutral losses as well, whereas original b and y ions, containing sugar moiety, are typically not detectable under CAD conditions. Therefore, peptide sequence can be reliably derived from pyrylium and furylium fragment ion series [291]. Moreover, characteristic immonium-related furylium and pyrylium derivatives of Amadori peptides at *m*/*z* 162.1 and 192.1 were successfully applied to development of a specific precursor ion scanning method, applicable for discovery of glycation sites in proteins [239]. Unfortunately, this approach is limited to monosaccharide-derived modifications, and is not applicable to lactose-derived early glycation products [240,292]. However, for ADP-ribose-derived glycation products, a characteristic loss of adenosine monophosphate (AMP) at *m*/*z* 348.08 could be observed [293]. In contrast to CAD, ECD or ETD techniques result in specific cleavage of the peptide backbone between amide nitrogen and Cα, whereas the side chain Amadori and Heyns moieties remain unaffected [239,294,295]. Thereby, the sequences of glycated peptides can be assigned by c- and z-ion series [289,296]. Finally, these observations with fragmentation of glycated peptides resulted in development of new analytical approaches, such as MSA experiments, comprising CAD- and ETD-based scans [52].

In contrast to Amadori and Heyns compounds, CAD-MS/MS spectra of AGE peptides dominate with the fragments, related to backbone cleavage (i.e., b and y ions) [242], although the side chains of some intermediates, such as CMA and CEA, are involved in fragmentation as well [266]. A pronounced backbone fragmentation results in reliable sequence assignment by fragmentation patterns and confident annotation of proteins by database analysis with proteomics search engines [32]. The annotations can be additionally validated by characteristic modification-specific signals in tandem mass spectra of AGE peptides [242,243]. Thus, the presence of imidazolone AGEs (Glarg and MG-Hs) in peptide sequence can be confirmed by abundant series of internal fragments, accompanied with less intense ammonia losses, which can be considered as diagnostic for the peptides containing basic heterocyclic AGEs [243]. Additionally, identity of these modifications can be confirmed by indicative signals at *m*/*z* 152.1 and 166.1, for Glarg- and MG-H, respectively [243]. These arginine immonium ion-related products of an intra-molecular SN-reaction [297] are related to the signal at *m*/*z* 112, known to be indicative for arginine-containing peptides [298]. It is worth mentioning, that these signals are present in the fragmentation patterns of glyoxal- and methylglyoxal-derived dihydroxyimidazolidines (G-DHI and MG-DHI), the precursors of Glarg and MG-H, respectively [243,260]. This allows their distinguishing from CMA and CEA modifications, isomeric to G-DHI and MG-DHI, respectively. For CML and CEL, known as the major lysine-derived AGEs in vivo [16,299], characteristic α-amino-ε-caprolactam and tetrahydropyridine immonium related ions were detected at the *m*/*z* 142.1 and 187.1 (CML) and 156.1 and 201.1 (CEL), respectively [242].

To summarize, peptide-based glycation models represent a powerful tool for dissection of glycation pathways and characterization of the products formed. It allows simulation of different glycation systems, such as food cooking [267,268], mammalian [51], or even plant [32] organisms. Accordingly, increasing application of these techniques in food chemistry, diagnostics, and plant biology can be expected.

### 3.2. Individual Glycation Sites in Human Proteins as the Markers of Diabetes Mellitus

As has been well-known since the late 1960s, the levels of glycated blood proteins correlate with the concentrations of glucose in plasma [300], although a clear evidence for a causative role of glycation for long term complications of metabolic diseases has not yet been reported [301]. Thus, hemoglobin isoform HbA_1c_, glycated at the N-terminal valine of its â chain, is a well-known diagnostic marker of diabetes mellitus (DM) [302]. Indeed, its only glycation site delivers reliable information about average blood glucose levels over approximately three months, being an important marker of a long-term glycemic control [302]. To address the changes in blood glucose profile over shorter periods of time, glycated HSA can be used as a marker. Accordingly, various spectroscopic, chromatographic and immunochemical methods for quantification of glycated HSA were established during the last decades [115,303]. Among them, immunoassays appear to be the most promising for diagnostics [34,304,305]. However, all clinically approved approaches deliver only global glycation rates, whereas individual lysyl residues are highly variable in their reactivities towards glucose [306]. At least to some extent, it can be explained by dependence of glycation levels from sequence [52] and structure [53,287,307] consensus moieties. Therefore, averaged abundance of all 58 potential HSA glycation sites, delivered by conventional techniques, might be less informative in comparison to quantification of glycation levels at individual lysyl residues [115]. Moreover, due to different half-lives of individual plasma proteins (varying 2–21 days [308,309,310,311,312,313,314]), individual glycation sites could provide information about the levels of blood glucose over any desired period of time. Hence, these approach might be able to deliver not only the information about efficiency of therapy as a glycemic control tool, but also to be a diagnostic marker, recognizing fluctuations of blood glucose levels during the onset of disease [315,316].

Indeed, Zhang et al. reported multiple glycation sites in the proteins of human plasma and erythrocyte membranes [52,137]. Thereby, the authors demonstrated higher numbers of glycated residues in the proteins obtained from T2DM and impaired glucose tolerance (IGT) patients (especially in erythrocyte membranes), in comparison to normoglycaemic controls. This fact might be related to the longer life span of red blood cells. Thereby, the analysis of glycation consensus motifs revealed alanine, valine, leucine and serine as the most common residues in close proximity to glycation sites [137]. Label-free quantification of the 18 most abundant Amadori peptides, detected in tryptic digests of T2DM and normoglycemic plasma, revealed higher abundances of some of them in diabetic patients, whereas two peptides were unique for disease [291]. This observation brought us to the assumption of possible DM biomarker properties of individual glycation sites.

**Figure 6:**
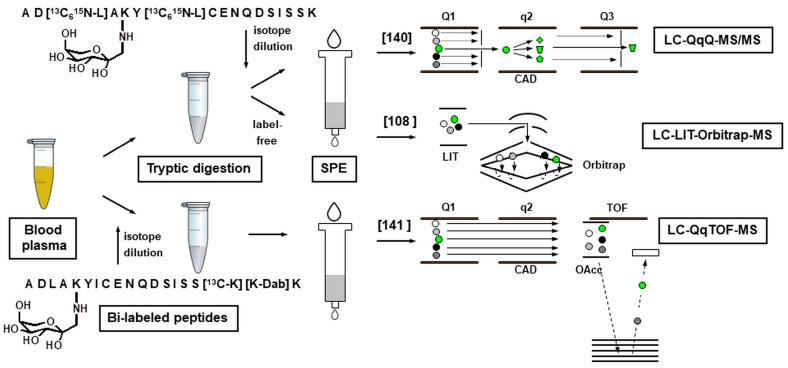
Quantification of individual protein glycation sites as Amadori-modified peptides in blood plasma tryptic digests by targeted and untargeted LC-MS approaches. LC-QqQ-MS/MS: green, white, black and grey circles are precursor ions, different shapes in green denote product ions; LC-LIT-Orbitrap-MS, LC-QqTOF-MS: green, white, black and grey circles denote quasi-molecular ions.

To proof this concept, we compared their abundances in small cohorts of T2DM patients and normoglycemic individuals [115]. For this, we established an untargeted workflow, relying on BAC, coupled off-line to RP-nanoUHPLC-HR-MS (Figure 6). Thereby, tandem mass spectra were acquired with an LTQ-Orbitrap-MS instrument operated in a DDA mode and searched against human database. After manual confirmation of all positive hits, label-free quantification was performed by integration of characteristic extracted ion chromatograms. By this procedure, glycation sites could be assigned to those: (i) found only in T2DM samples; (ii) significantly up-regulated in the diabetic group; and (iii) demonstrating no significant changes between groups, the first two of which represented prospective biomarkers [115].

To address the biomarker potential of the discovered candidates, we decided to switch to high-throughput absolute quantification. For this, we established two strategies based on a stable isotope dilution approach (Figure 6). The first one was based on ^13^C,^15^N-labeled glycated peptides, synthesized by glycation on solid phase [281], purified [284], and spiked to digested plasma prior to RP-HPLC-MS/MS analysis in multiple reaction monitoring (MRM) mode using two specific Q1/Q3 mass range combinations (transitions) [189]. Alternatively, internal standards were spiked directly to blood plasma, i.e., before tryptic digestion [174]. In this case, analysis relied on so-called bi-labeled dabsylated peptides [262], obtained by a solid phase peptide synthesis (SPPS) using the Fmoc strategy and pre-synthesized Amadori-modified building block [280]. Both strategies confirmed biomarker properties of the target peptides, and yielded comparable results. Remarkably, the efficiency of our proteomics-based strategy can be increased by simultaneous consideration of non-plasma biomarkers. Thus, Spiller and co-workers reported a combination of K141 of haptoglobin and HbA_1c_, which provided identification of diabetes with a sensitivity, specificity and accuracy of 94%, 98%, and 96%, respectively [317]. Recently, a new high-throughput LC-MS/MS MRM method for quantification of HSA glycation site at K_525_ was proposed [120]. The corresponding tryptic peptide ^525^KQTALVELVK is the most abundant species in plasma digests and can be quantified without enrichment. The authors highlighted a strong correlation of this single-site marker with HbA_1c_. Based on their data, the authors propose a 11% cut-off for the levels of glycated K_525_ in HSA, which increases the similarity of its behavior with HbA_1c_ [120].

Similar to early glycation, profiles of plasma AGEs were comprehensively addressed during last years. Recently, we have proposed precursor ion scanning methods for modification-specific signals (at *m*/*z* 187.1, 201.1, 152.1, 166.1 for CML, CEL, Glarg and MG-H, respectively) as a promising approaches for a comprehensive detection of AGE-modified sites in plasma proteins [242,243]. Thereby, the positive hits, discovered with this method were further evaluated by targeted DDA experiments, and sequence information for the peptides, containing specific AGEs, could be obtained. Applied to pooled T2DM plasma, this workflow revealed 21 carboxymethylation sites in 17 proteins including HSA [242]. Analogously, Schmidt and coauthors reported detection of 44 peptides, containing arginine-derived AGEs and representing 42 plasma proteins [243]. As some AGE-modified tryptic peptides (e.g., amide AGEs) can be considered as prospective T2DM biomarkers [19], precursor ion scanning for the characteristic immonium ion-related species might have a valuable diagnostic potential. Accordingly, the levels of *N^ε^*-(carboxymethyl)valine- and *N^ε^*-(carboxyethyl)valine-containing peptides of β-hemoglobin correlate well with severity of diabetes [184]. Moreover, recently five lysyl sites responsive to glycation (Amadori and CML modifications) were identified by targeted Sequential Window Acquisition of all Theoretical Mass Spectra (SWATH) analysis and confirmed as potential novel markers of diabetes [258]. Finally, Greifenhagen and coauthors reported LC-MS-based detection of 42 AGE modification sites in 22 high to medium abundant plasma proteins of diabetic patients, possible biomarker behavior of which needs to be characterized [234].

### 3.3. Proteomics in the Study of Anti-Glycative Defense

Most glycation pathways lead to generation of reactive dicarbonyl intermediates (e.g., GO, MGO, and 3-DG), enhanced formation of which ultimately causes dicarbonyl stress and related protein damage [318]. In agreement with this, the levels of á-dicarbonyls were shown to be increased in plasma and tissues of DM [319] and renal failure [320] patients. Expectedly, increased levels of MGO-derived protein modifications were also observed in ageing human tissues, for example, in lens of aged individuals [321]. Therefore, investigation of anti-Glycative defense pathways, such as glyoxalase system, becomes increasingly important when biology and biochemistry of in vivo tissue ageing is addressed.

The glyoxalase system plays a crucial role in glutathione (GSH) homeostasis both under normal and pathological conditions. It metabolizes reactive dicarbonyl compounds (RCCs), such as GO and, mostly, MGO, to less reactive and, hence, less harmful products [322]. Besides small amounts of reduced glutathione (GSH), it comprises two enzymes, namely glyoxalase 1 (Glo1) and glyoxalase 2 (Glo2), which catalyze conversion of MGO in *S*-d-lactoylglutathione and its subsequent cleavage with formation of d-lactate, respectively [323]. These enzyme activities prevent accumulation of reactive dicarbonyls in cells (especially under oxidative stress) and protect organism from development of carbonyl stress [324], thereby suppressing dicarbonyl-mediated glycation reactions [325] and playing a key role in cellular anti-glycation defense [326]. Accordingly, these enzymes can be expected to be protective in diseases, known to be accompanied with carbonyl stress [327].

Indeed, during the last decade, activity of glyoxalase pathway in presence of pathologies, such as DM and neurodegenerative disorders (e.g., Parkinson’s and Alzheimer’s diseases), and its influence on dicarbonyl proteome were studied comprehensively [327,328]. The term “dicarbonyl proteome” (DCP) was introduced by Rabbani and Thornalley to define collectively the proteins inactivated by MGO in physiological systems [329], for example, due to high MGO-dependent glycation levels in DM patients [323]. According to the currently available data, DCP includes at least albumin, hemoglobin, co-repressor protein sina3A, type IV collagen, áA lens crystallin, HIF1á (hypoxia-inducible factor 1á) co-activator protein p300, 20S proteasome subunits, mitochondrial proteins extracellular matrix proteins, lens crystallins and other high- and low-abundant proteins [326]. For example, according to the results of a comprehensive LC-ESI-HR-MS-based profiling, performed with cytosolic protein extracts of human endothelial cells, 344 of 1366 identified proteins contained MG-H or corresponding dihydroxyimidazolidine moieties [330].

It is important to note, that in the plant kingdom, glyoxalase system plays an important role as well. Thus, its impact in plant response to abiotic stress was clearly demonstrated in numerous proteomic studies [331]. For example, both activity and expression levels of Glo 1 demonstrated a stressor-dependent increase, when the mechanisms of salt tolerance of *Aeluropus lagopoides* (a halophyte C4 plant) were addressed by the bottom-up proteomic approach [332]. The similar effects could be observed under experimental drought conditions. For instance, application of differentially concentrated mannitol solutions to a basal part of rice leaf for 48 h resulted in induction of Glo1 expression [333]. Analogously, analysis of drought responsive proteome of sunflower leaves and leaf protein soluble fraction of wild watermelon demonstrated a similar alteration in the tissue levels of Glo1 product [334,335]. Heavy metal stress was also shown to induce expression of glyoxalases [331]. For example, application of high Cu(II) amounts to germinating rice seeds and roots revealed a strong up-regulation of both glyoxalases [336,337]. The same was observed when rice seeds were exposed to Cd(II) [338], although, in some cases, the activities of Glo1 and Glo2 were compromised [339], most probably, due to involvement of glutathione in phytochelatine biosynthesis. Interestingly, application of selenium attenuated this effect [339].

### 3.4. Glycation of Plant Proteins as the Marker of Ageing and Environmental Stress

Although AGEs are recognized as the markers of ageing, sub-clinical inflammation, and diabetic complications since several decades [340,341,342], their formation in plants was addressed only recently. Thus, in the beginning of the last decade, Sebekova and co-workers reported higher contents of AGEs in blood of vegetarians in comparison to omnivorous individuals [343]. Logically, this observation raised a question about formation of glycation products in raw plant-derived foods [344]. Further, this work was extended to in vivo glycation in plants, which was first reported by Thornalley and co-workers [345]. Currently, the phenomenon of protein glycation in plant organisms is being elaborated in four principal aspects: (i) molecular mechanisms of AGE formation; (ii) impact of AGEs in plant physiology; (iii) effect of glycation on nutritional value of plant-derived foods; and (iv) mechanisms and pathways behind anti-glycative defense [32,53,223].

First, the major methodological tool in plant glycation research was LC-MS/MS of exhaustive protein hydrolysates, established in Thornalley’s group since two decades [256]. Thus, using a panel of glycation and oxidation markers, Bechtold et al. confirmed the presence of glycated lysine and arginine residues in *Arabidopsis thaliana* proteins [345]. Thereby, for the first time, glycation was considered in the sense of plant response to environmental stress and proposed to be a factor of protein damage in plants, as it was earlier described for mammals [346]. However, in the context of plant biology, analysis of amino acid glycation adducts has some limitations. Indeed, it does not allow identification of individual proteins, serving as the targets of glycation, and specific glycation sites therein. However, as was earlier shown for mammals, this information is principle for understanding of physiological effects of glycation [60].

Nevertheless, this pioneer work opened an intensive discussion about a possibility of a diabetes-like state in plant tissue. Thus, Miyake and co-workers assume, that protein glycation in plants might be much more pronounced in comparison to mammals, due to much stronger persistent “hyperglycemia”, characteristic for these organisms [347,348,349]. Indeed, on the one hand, plant tissues contain a tremendous variety of carbohydrates, many of which are present in high abundances and/or are potent glycation agents [32,223]. On the other hand, due to intensive photosynthesis and respiration, the levels of oxidative processes are relatively high in plants [350]. Moreover, these organisms often encounter with environmental stress, accompanied with overproduction of reactive oxygen species (ROS) [351] and specifically hydroperoxides [352]. Therefore, high levels of lipid peroxidation [353] and monosaccharide autoxidation, i.e., the process of metal-catalyzed oxidation of sugars in presence of hydroperoxides [8], can be expected in plant systems. As both processes are accompanied with generation of á-dicarbonyls, plants might be subjected to a severe dicarbonyl stress [354,355] over the whole span of their life, and undergo intensive glycation. As both ROS generation and sugar accumulation are increased under stress conditions [175], the enhancement of glycation under high light stress conditions observed by Bechtold et al. [345] is in agreement with these considerations.

**Figure 7:**
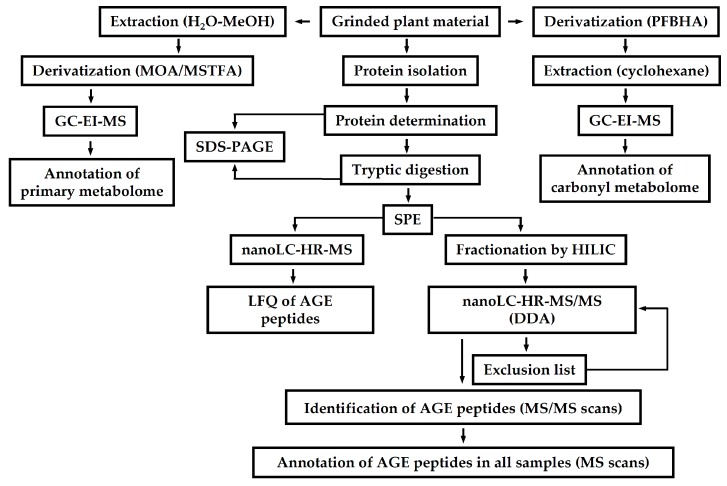
The experimental workflow for analysis of plant protein glycation and characterization of underlying mechanisms. The experiments relied on a combination of proteomics and metabolomics approaches.

At the next step, plant glycation patterns were addressed at the level of proteome. The approach, established in our group, relies on the bottom-up proteomic strategy and LC-MS and MS/MS analysis in data-dependent acquisition (DDA) mode. In the first line, we considered the patterns of the major AGEs, earlier identified in mammals and foods [251,252,253,261,274,285,356,357,358,359], and addressed the sources of their formation in plants using a combination of in vivo and peptide-based in vitro approaches [32]. For in vivo experiments, we established a workflow, comprising analysis of early and advanced glycation end products, as well as sugar profiling and dicarbonyl analysis (Figure 7). Thereby, for the first “proof of the concept” studies we considered only water-soluble proteins, while later we extended our analysis to the total proteome [223]. Relying on this combined strategy, we identified several specific features of plant glycation, clearly distinguishing it from the protein Maillard reaction in mammals. Thus, early glycated protein sites could not also be detected as AGE-modified, which might indicate a low impact of glycoxidation (Figure 1) in formation of AGEs. In contrast, based on the interpretation of glycation patterns in the context of the acquired metabolomic data (Figure 7), the autoxidative pathway seems to be the major route of AGE formation in plants [32]. Secondly, the fact that relatively few pyrraline sites were found, allows proposing non-oxidative pathway to be only a minor one. Finally, the numbers of plant amino acid residues involved in formation of AGEs were at least five times higher in comparison to the representation of Amadori and Heyns modification sites [32], which dramatically differs from, for example, human plasma [52,137,234,242,243].

Despite these differences, in respect of protein glycation, plants demonstrate clear similarities with mammals. Thus, recently, existence of glycation hotspots, described earlier in mammalian proteins [306,360], was confirmed in plants [53]. Hence, glycation can be considered as a universal marker of ageing, characteristic for both plant and animal kingdoms. Additionally, both qualitative and quantitative [223,345] AGE patterns were shown to be affected by environmental stress, as was earlier described for mammals [361]. However, the most of the questions, concerning plant protein glycation are still to be answered. Thus, the biological role of this process in plants is completely unknown. In addition, involvement of glycation in the mechanisms of senescence and regulation of plant development needs to be addressed. Taking into account high glycation levels, observed in plant organisms, characterization of anti-glycative protective mechanisms is another important aspect of the plant Maillard research [362,363].

## 4. Conclusions

During the recent decade, proteomics became one of the main tools in the protein Maillard research. Generally, although exhaustive enzymatic hydrolysis and subsequent analysis of glycation adducts by LC-MS/MS with stable isotope dilution became a gold standard in protein AGE analytics, the knowledge about exact glycation targets and particular modification sites therein seems to be critical for understanding the biochemical and physiological aspects of in vivo Maillard reaction. The main reason for this is a large body of accumulated data, clearly indicating a site-specific character of glycation. Indeed, identification of glycation hotspots both in animal and plant proteins, differential reactivity of individual protein residues towards sugars and dicarbonyls, positive correlation of glycation rates with ageing and stress response, biomarker behavior of strictly particular glycation sites, and existence of well-tuned enzymatic and non-enzymatic anti-glycative defense might indicate involvement of AGEs in regulation of vital processes in living organisms. Therefore, characterization of responses of various human cells to glycation will remain the mainstream of the medical Maillard research. However, understanding of the mechanisms underlying plant glycation and resistance of plants to continuously enhanced glycation levels in combination with adequate translational approaches might essentially improve treatment of glycation-related diseases. In this context, the role of proteomics in Maillard reaction research, as a method providing understanding of structure–function relationships, will continuously increase in future.

## Figures and Tables

**Table 1:** Digestion strategies used in analysis of protein glycation.

#	Object	Analyzed Adducts	Methodology							Ref.
			Technique	Protein Isolation	Denaturing Buffer or Detergent	Reduction Alkylation	Protease	Chromatographic System	MS	
1	HSA	Fru-Lys	cap-HPLC-MS	PEG 6000, affinity chromato-graphy	0.5 M *tris*-HCl, 2.75 mmol/L EDTA, 6 mol/L guanidine-HCl, pH 8.1	DTT/IA	trypsin	C18 A: 2% ACN, 0.1% aq. FA B: 98% aq. ACN, 0.1% FA	ESI-IT-TOF-MS	[83]
2	plasma proteins	Fru-Lys	nano-UHPLC-MS	-	1% (*w*/*v*) SDS	TCEP/IA	trypsin	RP, nanoAcquity UPLC BEH130 A: 0.1% aq. FA B: 0.1% FA in ACN	ESI-LTQ-Orbitrap	[115]
3	HSA	Fru-Lys	nano-HPLC-MS	-	76% acetonitrile	-	trypsin	RP, C18 A: 0.2% aq. FA B: 0.2% FA in ACN	ESI-QqTOF, ESI-QqQ	[120]
4	HSA	AGEs	HPLC-MS, MALDI-MS	-	-	DTT	trypsin	RP, C18 A: 0.1% aq. TFA B: 0.1% TFA in ACN	ESI-IT, MALDI-TOF	[156]
5	RNase	AGEs	HPLC-MS	-	0.1 mol/L MOPS buffer, 6 mol/L urea, 1 mmol/L EDTA	DTT	trypsin	RP, C18 A: 0.1% aq. TFA/FA B: 0.1% TFA/FA in ACN	ESI-QqTOF, ESI-QqQ	[157]
6	HSA	Fru-Lys	nano-HPLC-MS	centrifugal conc.	-	DTT/IA	chymo-trypsin	RP, C18 A: 0.1% aq. FA B: 0.1% FA in ACN	ESI-QqTOF	[146]
7	HSA	Fru-Lys	2D-nano-HPLC-MS	PEG 6000, affinity chromato-graphy	0.5 mol/L *tris*-HCl, 2.75 mol/L EDTA, 6 mol/L guanidine-HCl, pH 8.1	DTT/IA	Glu-C	RP, BetaBasic C18 A: 0.1% aq. FA/ 2% ACN, B: 98% ACN, 0.1% FA	ESI-IT-TOF	[83]
8	β-Lg	AGEs	UHPLC-MS	-	-	DTT (after hydrolysis)	Glu-C	RP, C18 A: 0.1% aq. FA B: ACN	ESI-QqLIT	[149]
9	insulin	Fru-Lys	MALDI-MS	-	4 mol/L urea	DTT/ IA	Glu-C	-	MALDI-TOF	[158]
10	HSA	Fru-Lys	MALDI-MS	-	6 mol/L guanidine-HCl, pH 8.5, 100 mmol/L ABC	DTT/IA	Glu-C	-	MALDI-TOF	[148]
11	HSA	Fru-Lys	MALDI-MS	-	6 mol/L guanidine-HCl, pH 8.5, 100 mmol/L ABC	DTT/IA	Lys-C	-	MALDI-TOF	[148]
12	HSA	AGEs	HPLC-MS, MALDI-MS	-	-	DTT	Lys-C	RP, C18 A: 0.1% aq. TFA B: 0.1% TFA in ACN	ESI-IT, MALDI	[156]
13	α-La, β-Lg	AGEs	MALDI-MS	-	-	DTT (after hydrolysis)	Asp-N	-	MALDI-TOF	[159]
14	plasma proteins	Fru-Lys	nano-HPLC-MS	BAC	8 mol/L urea, 0.5 mmol/L EDTA, 100 mmol/L ABC	DTT/IA	Arg-C	RP, C18 A: 0.2% aq. HOAc, or 0.05% aq. TFA or 0.1% aq. TFA B: 90% ACN	ESI-LIT	[160]
15	ubiquitin	Fru-Lys	cap-HPLC/off-line FIA-MS	-	-	-	pepsin	RP, C18 A: 0.1% aq. TFA B: 0.1% TFA in ACN	ESI-FT–ICR	[151]
16	HSA	AGEs	HPLC-MS	-	-	-	proteina-se K	RP, C18 A: 0.1% aq. TFA B: 0.1% TFA in ACN	ESI-QIT	[152]
17	IgG, plasma proteins	Fru-Lys	MALDI-MS	ultra-filtration	-	-	papain	-	MALDI-TOF	[153]

ABC, ammonium bicarbonate buffer; ACN, acetonitrile; Fru-Lys, *N^ε^*-(fructosyl)lysine; HOAc, acetic acid; IgG, immunoglobulin G; PEG, polyethylene glycol; TCEP, *tris*-(2-carboxyethyl)-phosphine hydrochloride.

## Abbreviations

2D-GE	two-dimensional gel electrophoresis
2-HNP	2(hydroxymethyl)pyrylium
3-DG	3-deoxyglucasone
3-DG-H	3-deoxyglucosone-derived hydroimidazolone
AALS	anionic acid labile surfactant
α-La	α-lactalbumin
ABC	ammonium bicarbonate buffer
ACN	acetonitrile
AGEs	advanced glycation end-products
ALEs	advanced lipoxidation end products
Apo-I	apolipoprotein I
β-Lg	β-lactoglobulin
BAC	boronic acid affinity chromatography
BSA	bovine serum albumin
BUP	bottom-up proteomic
CAD	collision-activated dissociation
CE	capillary electrophoresis;
CEA	*N^δ^*-(carboxyethyl)arginine
CEL	*N^ε^*-(carboxyethyl)lysine
CMA	*N^δ^*-(carboxymethyl)arginine
CML	*N^ε^*-(carboxymethyl)lysine
CZE	capillary zone electrophoresis
DCP	dicarbonyl proteome
DDA	data-dependent acquisition
DHX	deuterium–hydrogen exchange
DIA	data-independent acquisition
DIGE	difference gel electrophoresis
DM	diabetes mellitus
DR	double resonance
DTE	dithioeritritol
DTT	dithiothreitol
ECD	electron capture dissociation
EI	electron (impact) ionization
ESI	electrospray ionization
ETD	electron transfer dissociation
EXC	cation exchange chromatography
FA	formic acid
FIA	flow injection analysis
Fmoc	9-fluorenylmethoxycarbonyl
FT-ICR	Fourier transform-ion cyclotron resonance
Fru-Lys	*N^ε^*-(fructosyl)lysine, Amadori compound
GC	gas chromatography
G-DHI	glyoxal-derived dihydroxyimidazolidine
GELFrEE	gel-eluted liquid fraction entrapment electrophoresis
Glarg	1-(4-amino-4-carboxybutyl)-2-imino-5-oxo-imidazolidine, glyoxal-derived hydroimidazolone
Glo1	glyoxalase 1
Glo2	glyoxalase 2
GO	glyoxal
GOLD	glyoxal-derived lysine dimer
GPF	gas phase fractionation
GSH	glutathione
HbA	hemoglobin A
HbA_1C_	glycated hemoglobin
HIF1α	hypoxia-inducible factor 1α
HILIC	hydrophilic interaction liquid chromatography
HOAc	acetic acid
HPD	high pressure denaturation
HPLC	high-performance liquid chromatography
HR	high resolution
HR-MS	high resolution mass spectrometry
HSA	human serum albumin
IA	iodoacetamide
IgG	immunoglobulin G
IGT	impaired glucose tolerance
IMAC	immobilized metal affinity chromatography
IP-RP-HPLC	ion-pair reversed-phase high performance liquid chromatography
IT	ion trap
LC	liquid chromatography
LC-MS/MS	liquid chromatography-tandem mass spectrometry
LC-UV	liquid chromatography with ultraviolet detection
LIT	linear ion trap
MALDI	matrix assisted laser desorption/ionization
MG-DHI	methylglyoxal-derived dihydroxyimidazolidine
MG-H1	*N^δ^*-(5-methyl-4-oxo-5-hydroimidazo-linone-2-yl)ornithine, methylglyoxal-derived hydroimidazolone 1
MGO	methylglyoxal
MOA	methylhydroxylamine hydrochloride
MRM	multiple reaction monitoring
MS	mass spectrometry
MS/MS	tandem mass spectrometry
MSA	multi-stage activation
MSTFA	*N*-methyl-*N*-(trimethylsylil)trifluoroacetamide
nanoLC	nano-scaled liquid chromatography
NLMS3	neutral loss triggered MS3
oPDA	*o*-phenylenediamine
PEG	polyethylene glycol
PTMs	post-translational modifications
q	RF-only quadrupole
Q	quadrupole mass analyzer
QIT	quadrupole ion trap
QqQ	triple quadrupole
QqTOF	quadrupole-time of flight
RAGEs	receptors to advanced glycation end products
RCCs	reactive dicarbonyl compounds
RI	ribonuclease inhibitor
RNase A	ribonuclease A
ROS	reactive oxygen species
RP-HPLC	reversed-phase high performance liquid chromatography
RP-SPE	reversed phase-solid phase extraction
SDS	sodium dodecyl sulfate
SDS-PAGE	polyacrylamide gel electrophoresis is sodium dodecyl sulfate
SELDI	surface-enhanced laser desorption/ionization
SPE	solid phase extraction.
SPPS	solid phase peptide synthesis
SWATH	Sequential Window Acquisition of all Theoretical Mass Spectra
T2DM	type 2 diabetes mellitus
TCEP	*tris*-(2-carboxyethyl)-phosphine hydrochloride
TDP	top-down proteomics
THP	tetrahydropyrimidine
TOF	time of flight
UHPLC	ultra-high performance liquid chromatography
*v*/*v*	volume/volume
